# Snapping scapula syndrome: pictorial essay

**DOI:** 10.1590/0100-3984.2017.0226

**Published:** 2019

**Authors:** Stefane Cajango de Carvalho, Adham do Amaral e Castro, João Carlos Rodrigues, Wagner Santana Cerqueira, Durval do Carmo Barros Santos, Laercio Alberto Rosemberg

**Affiliations:** 1 Hospital Israelita Albert Einstein, São Paulo, SP, Brazil.; 2 A.C.Camargo Cancer Center, São Paulo, SP, Brazil.

**Keywords:** Scapula, Joint diseases/diagnostic imaging, Shoulder, Escápula, Doenças articulares/diagnóstico por imagem, Ombro

## Abstract

Snapping scapula syndrome manifests as an audible or palpable crackling during the sliding movements of the scapula over the rib cage, often perceived during physical or professional activities. It can be caused by morphological alteration of the scapula and rib cage, by an imbalance in periscapular musculature forces (dyskinesia), or by neoplasia (bone tumors or soft tissue tumors). In this pictorial essay, we review the main causes of snapping scapula syndrome, exemplified by a collection of didactic cases.

## Abstract (pt)

A síndrome da escápula em ressalto manifesta-se como uma crepitação audível ou palpável durante os movimentos de deslizamento da escápula sobre o gradil costal. Frequentemente percebida em atividades físicas ou profissionais, as suas causas podem ter origem na alteração morfológica da escápula e gradil costal, no desequilíbrio de forças da musculatura periescapular (discinesia) ou ainda em tumores ósseos ou de partes moles. O presente estudo revisou de forma ilustrativa as principais causas da síndrome da escápula em ressalto, exemplificadas por meio de uma coletânea de casos didáticos.

## DEFINITION AND EPIDEMIOLOGICAL ASPECTS

Snapping scapula syndrome is defined as an audible or palpable clicking of the scapula during movements of the scapulothoracic joint^(^^1^^)^. It typically affects young, active patients, who often report a history of pain, resulting from overuse, during rapid shoulder movements or during sports activities^(^^2^^)^. These symptoms can have insidious onset, can occur after a change in the pattern of physical activity, or can be associated with trauma^(^^3^^)^.

## ANATOMY AND BIOMECHANICS

The scapula is a flat, triangular bone that lies between the second and seventh ribs. As previously described^(^^3^^-^^5^^)^, it has two surfaces (ventral and dorsal), three borders (superior, lateral, and medial), and three angles (superomedial, inferomedial, and lateral).

**Figure 1:**
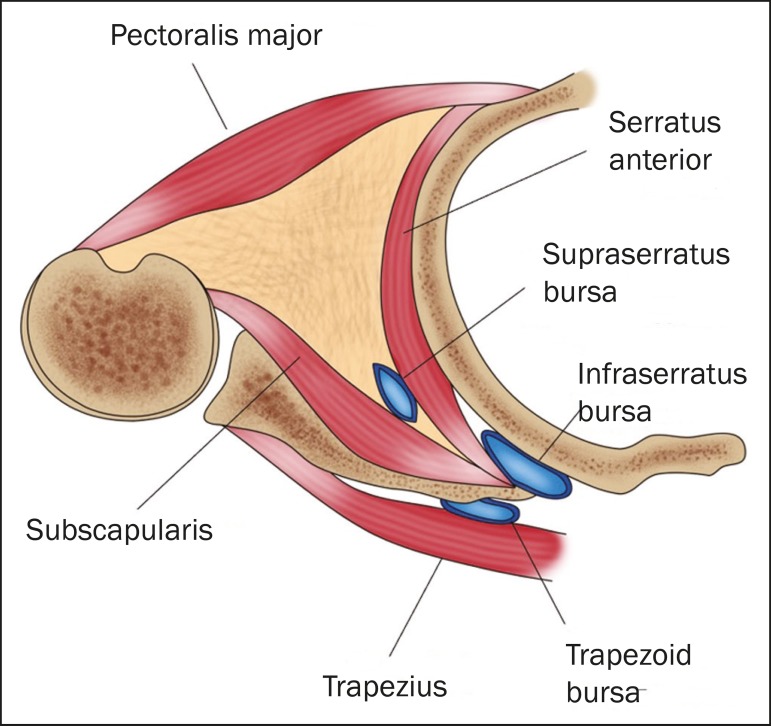
Schematic representation of the musculature and bursae involved in snapping scapula syndrome.

**Figure 2:**
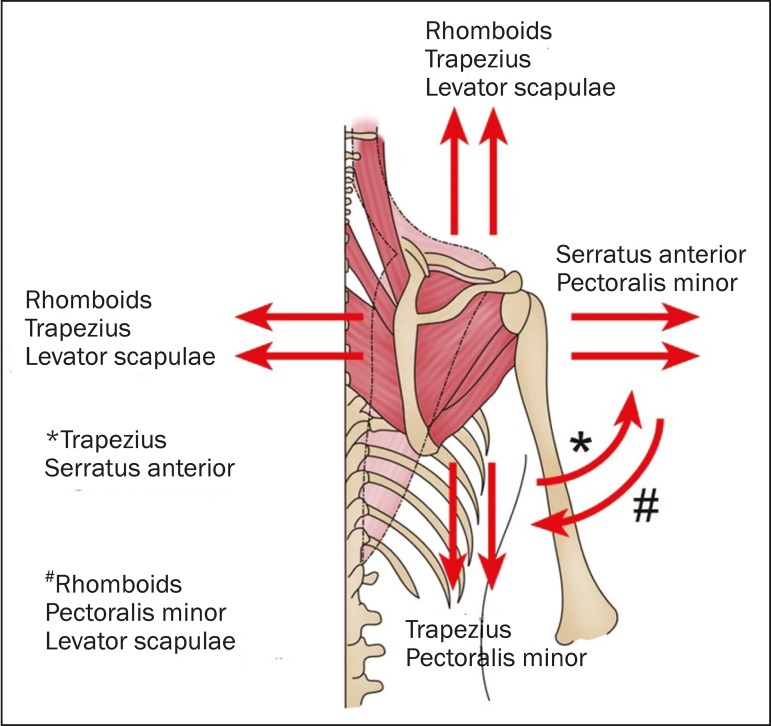
Schematic representation of the biomechanical vector of the musculature involved in scapular movement. The upper and lower portions of the trapezoid are shown in pink, and the central portion is translucent, demarcated by the dotted line.

**Figure 3:**
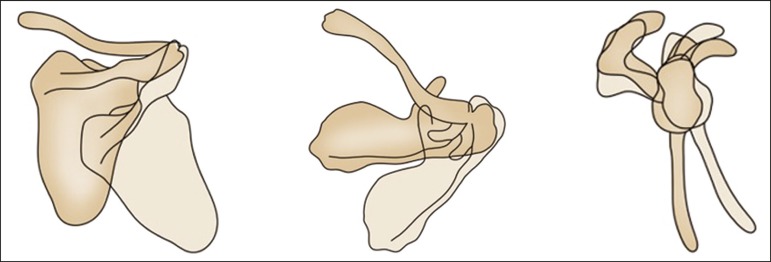
Schematic representation of the movement of the scapula, showing, from left to right, abduction/adduction, rotation, and upward/downward movements.

**Figure 4:**
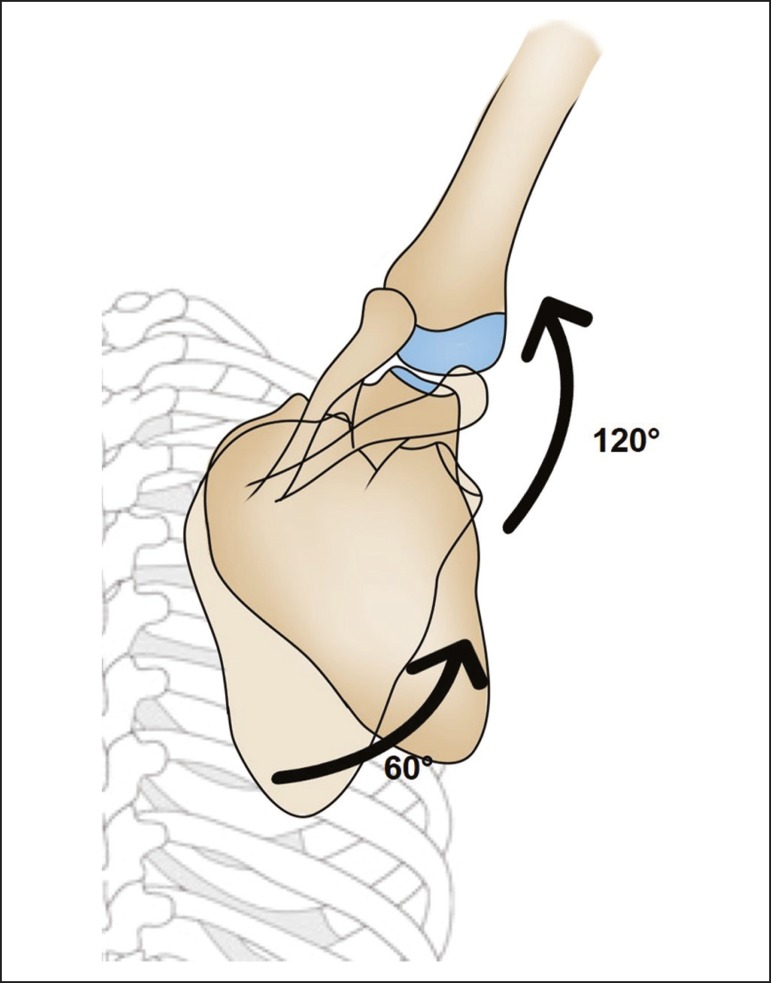
Schematic representation of the 2:1 scapulohumeral rhythm. For example, during a 180° abduction of the arm, 60° are achieved by rotation of the scapula and 120° are achieved by rotation of the humerus.

The articulation between the scapula and the rib cage is one of the most incongruous in the human body, because it does not have true joint structures but rather is surrounded by a complex of muscles, which is divided into three layers: superficial, intermediate, and deep. The superficial layer comprises the trapezius and latissimus dorsi muscles, which can be accompanied by a bursa located between the inferomedial angle and superficial fibers of the latissimus dorsi muscle^(^^3^^)^. The intermediate layer consists of the major rhomboid, minor rhomboid, and levator scapulae muscles. The trapezoid bursa lies between the trapezius muscle and the base of the shoulder blade. The deep layer consists of the serratus anterior and subscapularis muscles, containing the infraserratus bursa, located between the serratus anterior muscle and the rib cage, and the supraserratus bursa, located between the serratus anterior and subscapularis muscles^(^^3^^)^. Figure 1 illustrates the bursae and their respective anatomical relationships. The control and proper positioning of the scapula are fundamental for the correct functioning of the glenohumeral joint. During normal shoulder movement, the scapula needs to be properly aligned in multiple planes of motion, a situation that depends on harmonic and synchronous actions between the various scapular muscles. The scapula receives different combinations of forces exerted by the muscles inserted therein, producing movements of abduction, adduction, elevation, depression, and rotation (Figures 2 and 3). There is an arc-of-motion pattern between the glenohumeral joint and the scapulothoracic joint, known as the scapulohumeral rhythm, which has a 2:1 ratio. In other words, for every two degrees of movement of the humerus, the scapula moves one degree^(^^6^^)^, as depicted in Figure 4.

## ANATOMICAL VARIATIONS AND DISEASES THAT CAN CAUSE THE SYNDROME

### Superomedial angle of the scapula and anatomical variations

**Figure 5:**
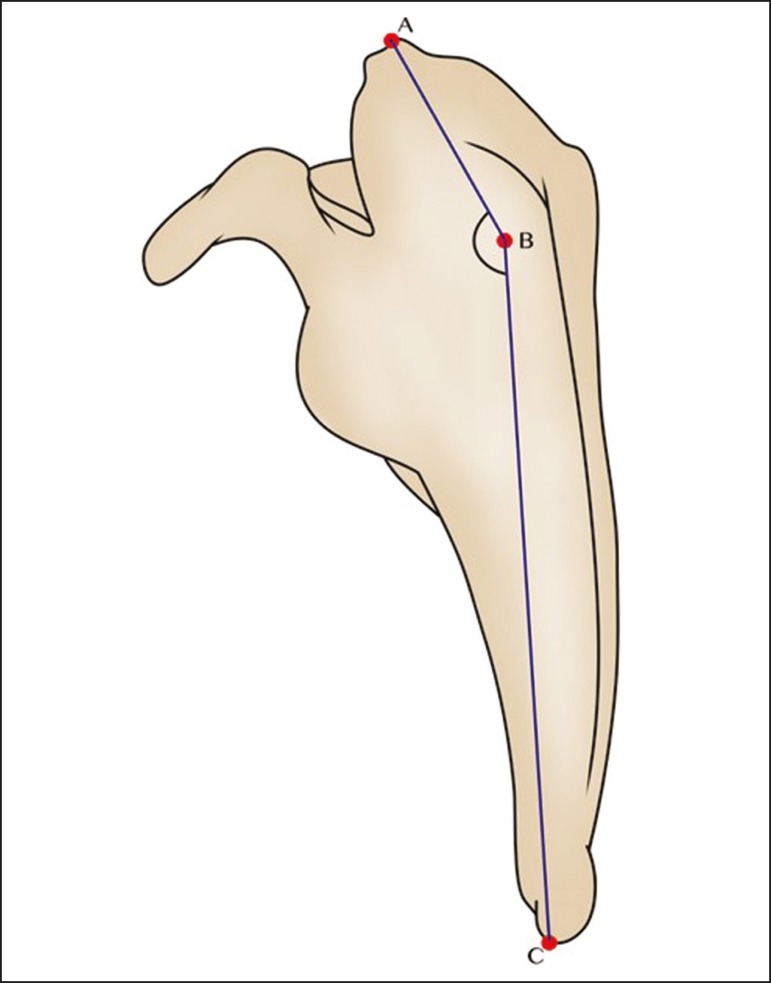
Schematic representation of the costal surface of the right scapula, showing the ABC measurement of the superomedial angle.

**Figure 6:**
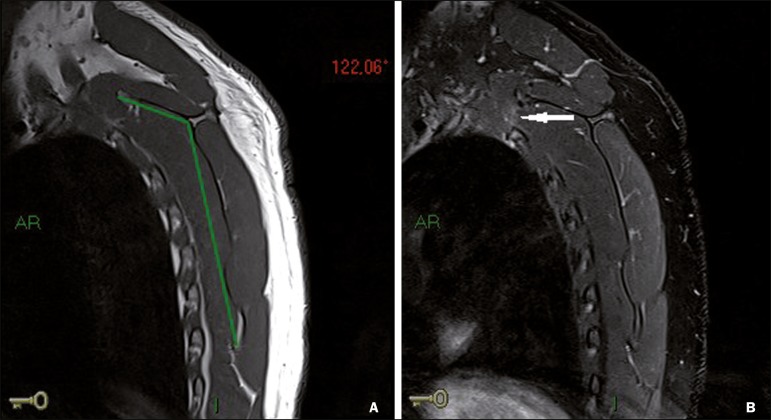
A 42-year-old male patient with a 7-year history of intermittent left-sided scapular pain, accompanied by snapping. The patient had been swimming, walking, and cycling on a regular basis. Sagittal MRI of the left scapula, with fat-saturated T1- and T2-weighted sequences (**A** and **B**, respectively), showing a 122° reduction in the superomedial angle of the scapula (the black lines in **A** indicate how the angle is measured), with a consequent reduction in the space between the second rib and the superior border of the scapula. Mild muscular edema and slight edema of the adjacent (second) rib (arrow in **B**).

**Figure 7:**
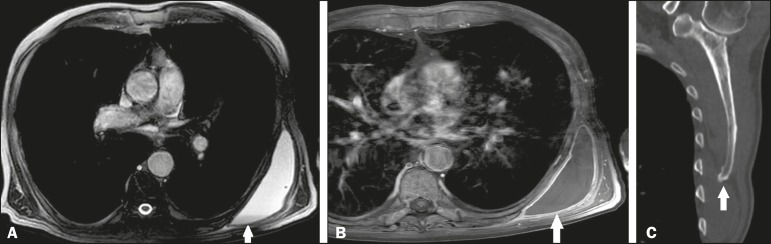
An 87-year-old male patient complaining of bulging of the posterolateral thoracic wall. The patient had been swimming on a regular basis. Contrast-enhanced axial MRI of the left scapula, with fat-saturated T1- and T2-weighted sequences (**A** and **B**, respectively), showing fluid distention and parietal enhancement of the infraserratus bursa (arrows) with a fluid-fluid level (arrow in **A**), due to a bone projection in the inferomedial angle of the scapula, as shown on a CT scan (arrow in **C**).

The scapulothoracic joint is cushioned by the serratus anterior and subscapularis muscles, as well as by the bursae^(^^7^^)^. The superomedial angle, inferomedial angle, and medial border of the scapula are relatively less protected by underlying muscles and bursae, and the upper medial border and lower pole exhibit wide anatomical variability^(^^4^^,^^7^^)^. When no obvious deformity is found, one should look for anatomical variations, such as an anomalous anterior curvature of the superomedial angle of the scapula, which is considered one of the main causes of the syndrome. The superomedial angle of the scapula has been measured in anatomical specimens and found to range from 124° to 162° (mean, 144.34 ± 9.09°)^(^^7^^)^; when the angle is lower than 142°, the chances of scapular snapping increase^(^^8^^)^. The superomedial angle is measured on the anterior surface of the scapula, with three anatomical reference points (Figures 5 and 6): the superior angle, the spine, and the inferior angle. A bone projection at the lower pole is the second most common site for symptoms^(^^4^^)^ (Figure 7).

### The Luschka tubercle

The Luschka tubercle is a hook-shaped bony protuberance, located at the upper medial border of the scapula, which can reduce the space between the scapula and the rib cage and be a predisposing factor for scapular snapping^(^^7^^)^.

### Scapular dyskinesia, insufficiency of the serratus anterior muscle, and injury of the long thoracic nerve

**Figure 8:**
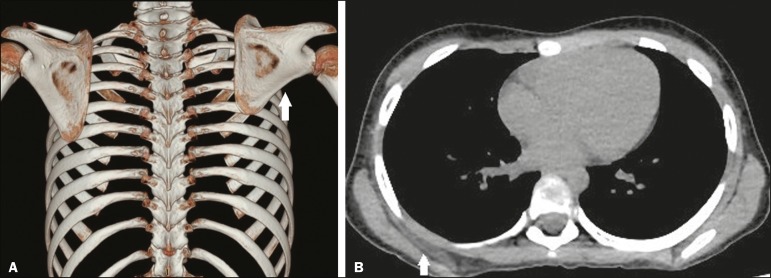
A 9-year-old female patient with a 1-month history of elevation of the right scapula, unrelated to pain, trauma, or surgery. **A:** Three-dimensional CT reconstruction of the rib cage showing winging of the right scapula (arrow). **B:** Axial CT scan, with a soft-tissue window setting, showing denervation with atrophy and fatty replacement of the serratus anterior muscle (arrow), showing the contralateral side for comparison. There was no evidence of extrinsic compression of the long thoracic nerve.

**Figure 9:**
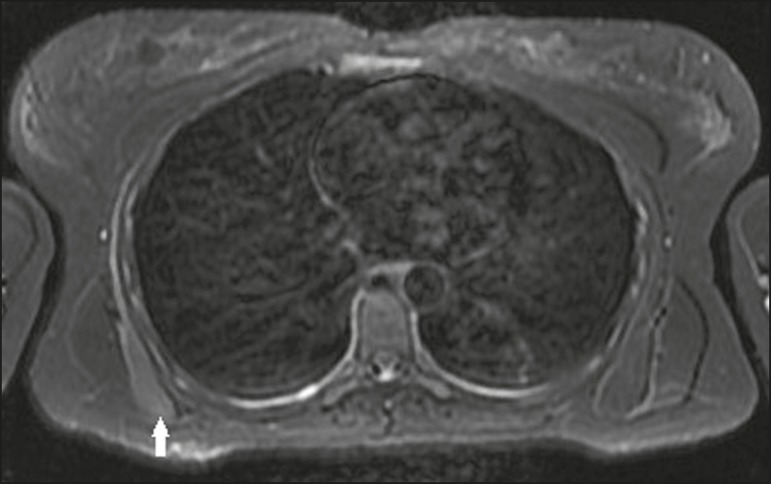
A 32-year-old female patient with a 3-month history of shoulder pain and scapular asymmetry. The patient had been running on a regular basis. Axial T2-weighted fat-saturated MRI scan of the scapulae showing denervation and edema of the serratus anterior muscle (arrow), without significant atrophy. There was no evidence of extrinsic compression of the long thoracic nerve.

Scapular dyskinesia, a common clinical finding, is defined as abnormal movement, positioning, or function of the scapula during shoulder movement. It can be the cause or consequence of many forms of shoulder pain and dysfunction. There are multiple causes of dyskinesia. Articulatory causes include acromioclavicular joint arthrosis, glenohumeral joint instability, and glenohumeral joint disorder. Musculoskeletal causes include thoracic kyphosis and nonunion of a clavicular fracture, as well as shortening, rotation, or angulation of the clavicle. Neurological causes include paralysis of the long thoracic nerve, paralysis of the eleventh cranial nerve, and cervical radiculopathy^(^^9^^)^, as depicted in Figures 8 and 9. The most common mechanisms involve imbalances of the intrinsic musculature, with inflexibility or inhibition of normal muscle activation^(^^9^^)^. Scapular snapping can be present in dyskinesias, because the abnormal movements bring the extremities of the scapula into closer proximity to the rib cage. Regardless of the cause of dyskinesia, the final result in most cases is a scapula in pronation, which is not conducive to optimal shoulder function and results in subacromial space reduction with symptoms of impingement^(^^9^^)^.

### Sequelae of fractures of the scapula and rib cage

The sequelae of fractures of the scapula and rib cage can cause bone deformities. Such deformities can increase the friction among the structures of the scapulothoracic joint^(^^10^^)^.

### Bursitis

**Figure 10:**
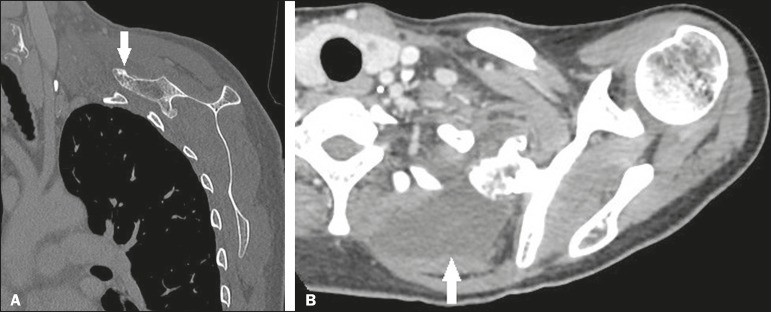
A 46-year-old female patient, in follow-up for osteochondroma for 8 years and presenting with a 7-month history of constant pain. CT of the scapula, in coronal and axial slices (**A** and **B**, respectively), demonstrates pedunculated osteochondroma in the anterior superior aspect of the scapula, in close proximity to the posterior border of the first and second ribs on the left (arrow in **A**). Marked fluid distention in the region of the supraserratus bursa (arrow in **B**).

Scapulothoracic bursitis can occur after a single traumatic insult, as a result of repetitive movements of the scapulothoracic joint, or as a result of scapular dyskinesia. Abnormal scapular movement can be caused by overuse of the muscles, muscle imbalance, or pathological conditions of the glenohumeral joint^(^^3^^)^. When the muscles of the costal surface of the scapula decrease in size, the scapula rotates forward, coming into closer proximity to the rib cage, generating friction with the chest wall during movement, causing inflammation in the scapulothoracic space^(^^3^^)^, as shown in Figures 7 and 10.

### Bone tumors

**Figure 11:**
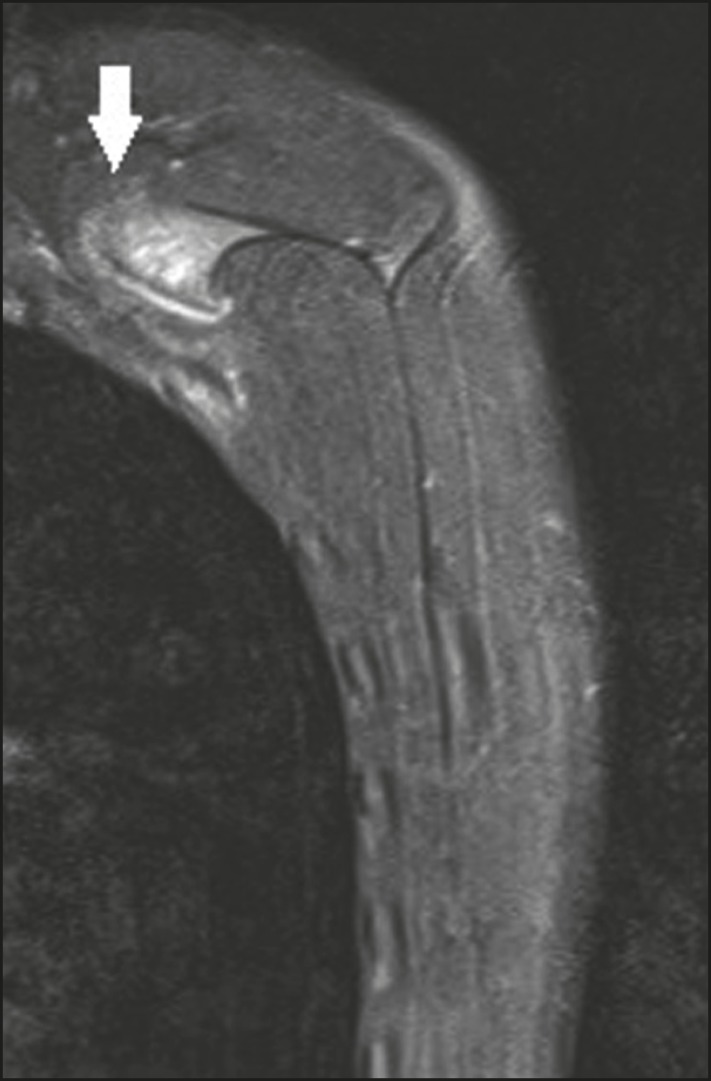
A 14-year-old male patient with a 6-month history of bulging and discomfort in the left scapula. The patient had been playing water polo on a regular basis. MRI of the left scapula, with a sagittal slice and a fat-saturated T2-weighted sequence, showing osteochondroma in the superomedial angle of the scapula, with a thin cartilaginous layer, invading the space between the first and second ribs (arrow). Edema of the musculature between the osteochondroma and the rib cage, suggesting friction.

**Figure 12:**
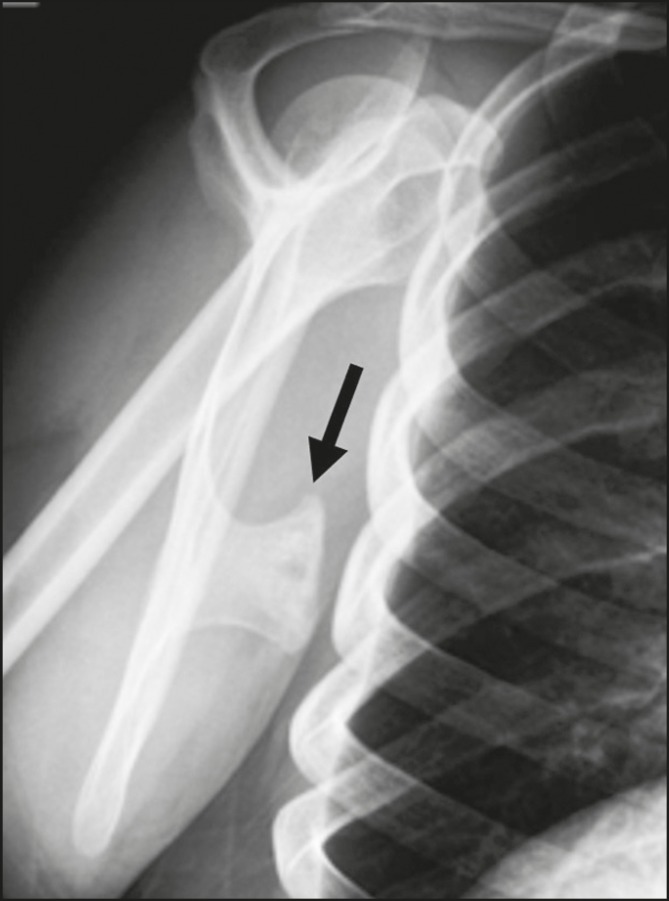
An 18-year-old male patient with pain in his right arm. X-ray showing broad-based exostosis in the lower third of the scapular body (subscapular fossa, arrow).

Osteochondroma, also known as exostosis, is the most common benign primary bone tumor of the scapula, being solitary in approximately 90% of cases and multiple, in the form of hereditary multiple exostoses, in approximately 10%. Such tumors are considered alterations of the growth plate, specifically its failure to increase in size during skeletal maturation^(^^11^^)^. They usually involve the metaphysis of long bones and, more rarely, the scapula (in 4-6% of cases). An osteochondroma can be symptomatic, mainly due to its mass effect, creating the appearance of scapular winging, together with crackles, and altering the scapulothoracic movement. It can also cause neurovascular compression, fractures, inflammation of the bursa, or malignant transformation^(^^11^^)^ (Figures 10-12). Although scapular chondrosarcoma is rare, the scapula is the second most common site of involvement of this disease, especially among men between 40 and 70 years of age^(^^3^^,^^4^^)^.

### Elastofibromas

**Figure 13:**
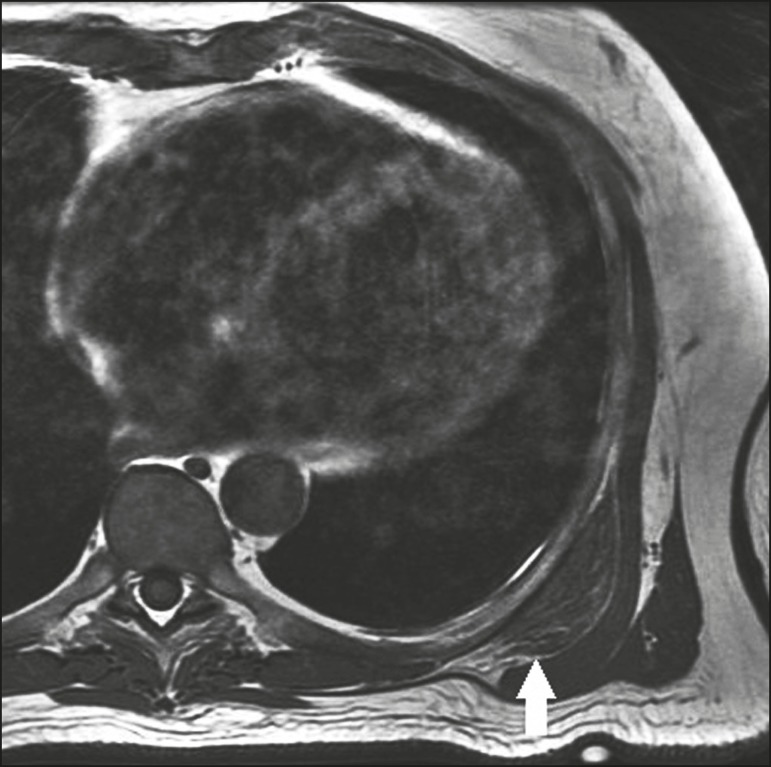
A 69-year-old male patient with a 90-day history of bulging, snapping, and pain in the left scapular region. The patient had engaged in weightlifting on a regular basis. Axial T1-weighted MRI of the left scapula, showing an elastofibroma deep within the serratus anterior muscle, interposed between the rib cage and the inferior angle of the scapula (arrow).

**Figure 14:**
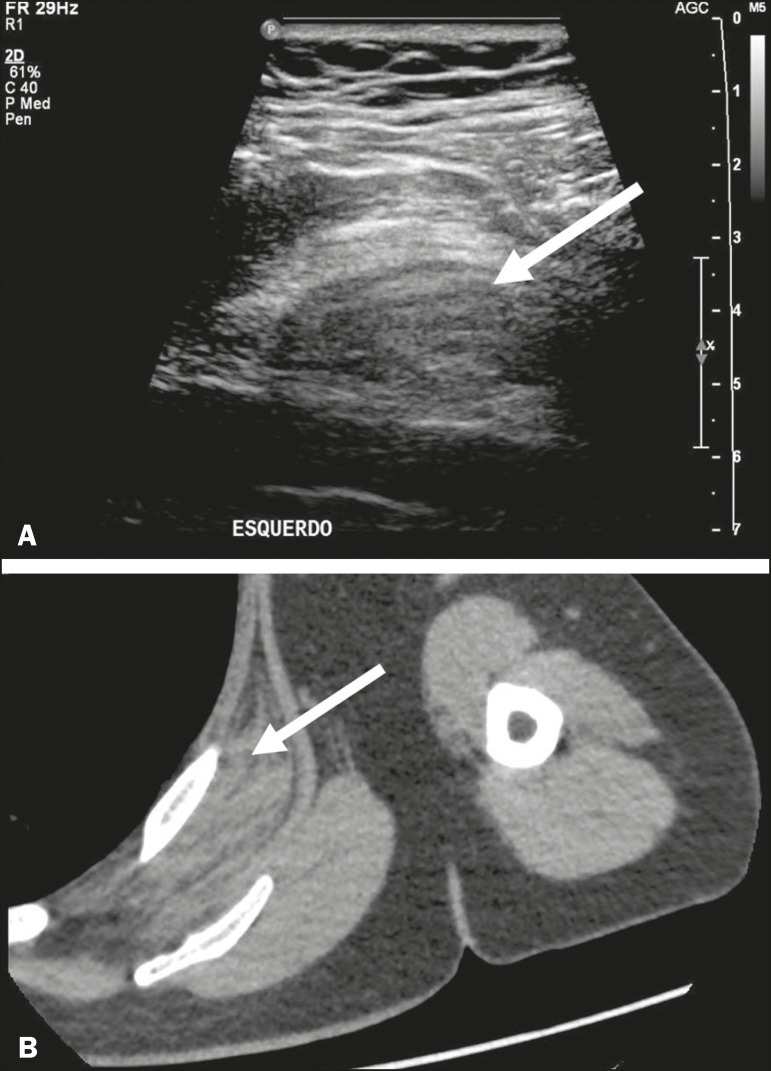
A 55-year-old male patient with increased volume in the left scapular region. Ultrasound (**A**) showing a predominantly hypoechoic, heterogeneous formation (arrow), located between the scapula and the rib cage. On CT (**B**), the formation presents soft-tissue density and fibrofatty striae, with a well-defined location between the costal grating and the ventral portion of the anterior serratus muscle, at the subscapular and infrascapular level, consistent with dorsal elastofibroma.

An elastofibroma is a benign soft tissue tumor with slow growth and a prevalence rate of up to 24% in the elderly, being most common among women between 55 and 70 years of age. Elastofibromas are believed to occur in response to repetitive microtrauma caused by friction between the scapula and the chest wall. An elastofibroma is typically located at the lower pole of the scapula, deep within the serratus anterior and latissimus dorsi muscles. It can manifest as an increase in subscapular or infrascapular volume, moderate discomfort or pain, crackles, clicking (snapping), or a blocked scapula^(^^12^^)^, as depicted in Figures 13 and 14.

## CONCLUSION

Although snapping scapula syndrome is rare, it can cause severe pain and functional limitation. Therefore, radiologists should be able to recognize its imaging findings. In this pictorial essay, we have illustrated the main causes of the syndrome, using imaging examinations.
